# Influence of Heating Rate on the Structure and Mechanical Properties of Aromatic BPDA–PDA Polyimide Fiber

**DOI:** 10.3390/polym12030510

**Published:** 2020-02-27

**Authors:** Wenke Yang, Fangfang Liu, Hongxiang Chen, Xuemin Dai, Wei Liu, Xuepeng Qiu, Xiangling Ji

**Affiliations:** 1 School of Materials Science and Engineering, Qilu University of Technology, Shandong Academy of Sciences, Jinan 250353, China; wkyang@qlu.edu.cn; 2 Polymer Composites Engineering Laboratory, Changchun Institute of Applied Chemistry, Chinese Academy of Sciences, Changchun 130022, China; 3 State Key Laboratory of Polymer Physics and Chemistry, Changchun Institute of Applied Chemistry, Chinese Academy of Sciences, Changchun 130022, China

**Keywords:** polyimide, polyimide fibers, polyamic acid, polyamic acid fibers, imidization

## Abstract

Aromatic polyimide fibers (PI) are usually produced in two steps. The precursor fibers of polyamic acid (PAA) are fabricated first, and then the fabricated fibers are converted into PI fibers through thermal treatment. In the second step (thermal treatment), the mechanical properties of the obtained PI fibers are remarkably affected. Here, the PAA fibers derived from *3,3’,4,4’*-biphenyltetra-carboxylic dianhydride and p-phenylenediamine are fabricated by a dry-jet wet-spinning method. Then, the PI fibers are prepared by heating PAA fibers from room temperature to 300, 350 and 400 °C under different heating rates, ranging from 1 °C/min to 80 °C/min. When the heating rate is low, the crystallization lags behind the imidization process, and begins only when the imidization degree reaches a high level. As the heating rate increases, the crystallization tends to occur simultaneously with the imidization process, and the degree of crystallinity of the PI fibers also greatly increases. Our findings suggest that a high heating rate causes the polymer chains to undergo high mobility during thermal treatment. The tensile modulus of the PI fiber further demonstrates a high dependence on the heating rate. Moreover, a short annealing process after treatment proves to be efficient in releasing residual stress and improving tensile strength.

## 1. Introduction

Aromatic polyimide (PI) fiber is a high-performance material with numerous outstanding properties. This fiber type can be applied in many fields, given its role in ensuring good thermal stability and its notable mechanical properties. Commercialized PI fibers such as P84^®^, Yilun^®^, Suplon^®^ and Shilon^®^ are widely used in high-temperature filtration and thermal protection, and have a potential market in high-tech applications, such as the use of military armors, radomes and spacecraft. The production of PI fibers was previously a direct result of spinning PI solutions. However, poisonous solvents such as m-cresols are frequently used in the construction of spinning dopes, thus seriously confining the production of PI fibers. Nowadays, nearly all kinds of commercialized PI fibers are produced via a two-step method. Polyamic acid (PAA), the precursor of polyimide, is first fabricated into fibers. Then, PI fibers are obtained through the imidization of PAA fibers via a thermal treatment. By means of this two-step process, PI fibers can be fabricated with the common solvents of *N,N′*-dimethylacetamide (DMAc), *N,N′*-dimethylformamide or *N*-methyl pyrrolidone.

From the viewpoint of PI fibers, producing stronger fibers is always the initial and final objective, as mechanical properties are the most important parameters for this kind of material. In general, the mechanical properties of PI fibers can be affected by various aspects, including spinning parameters, chemical structures and imidization conditions. 

As the precursor of PI fibers, PAA fibers are always fabricated via a wet-spinning or a dry-jet wet-spinning method. The conditions of the spinning process greatly influence the cross-section shape, surface and inside structures of the fiber, and can even determine whether the fiber can be successfully molded. As such, the optimization and adjustment of the spinning parameters based on the requirements of actual production are effective ways to promote the material’s mechanical properties. Several investigations have been conducted on the spinning parameters, including the spinning dope, coagulation bath, spinning speed and draw ratio.

Altering the chemical structure of PI fibers is another effective way of influencing the mechanical properties mentioned above. Compared with aliphatic monomers, the use of aromatic monomers to construct PI fibers always achieves outstanding mechanical properties. Moreover, as some heterocyclic diamine monomers are copolymerized into PI chains, additional intra/intermolecular interactions or improved ordered chain stacking in the fibers can significantly promote the mechanical properties of PI fibers. Heterocyclic diamine monomers such as 5-anino-2-(2-hydroxy-4-aminobenzene)-benzoxazole [1], 2-(4-aminophenyl)-5-aminobenzimidazole [2,3,4], 2-(4-aminophenyl)-6-amino-4(3H)-quinazolinone [5,6] and 5-amino-2-(4-aminobenzene)benzoxazole [1,3] are reportedly extremely effective in improving mechanical properties.

The imidization process greatly influences the mechanical properties of PI fibers, but the related studies remain far from adequate. During the imidization process, the sub-processes of solvent evaporation [7,8,9], chemical conversion [10,11,12], chain orientation [13,14] and crystallization [15] occur concurrently, and affect the final properties of PI materials. Cakmak [8] studied the relaxation and orientation of polymer chains in PI films by means of a real-time instrument, and found that the competition of the two events was affected independently at each stage of the imidization process. Hasegawa [16,17,18] investigated spontaneous molecular orientation in thermal imidization and discussed the role of driving force under different conditions. Liu et al. [19] studied PI fibers containing benzimidazole units, and found that H-bonding interactions are weakened and a sudden crystallization behavior occurs when the annealing temperature reaches 400 °C. This phenomenon is meaningful in promoting the mechanical properties of PI fibers.

In our previous study, we investigated the imidization conversion of typical PI fibers with *3,3’,4,4’*-biphenyltetra-carboxylic dianhydride (BPDA) and p-phenylenediamine (PDA) under different thermal treatment conditions. We found that crystallization lags behind the imidization reaction, and is affected significantly by heating procedures. PI fibers obtained via sudden thermal treatment exhibit higher crystallinity and greater tensile modulus compared with samples obtained via slow ramp heating at 5 °C/min; this difference originates from the difference of heating rate. Our previous finding further indicates that the heating rate is a crucial factor in the thermal imidization process of PI fibers.

In this study, we systematically investigate the influence of the heating rate on the thermal imidization process. The same batches of PAA fibers are treated under different heating rates, ranging from 1 °C/min to 80 °C/min. Imidization conversion and crystallization are traced and characterized by thermogravimetric analysis (TGA), dynamic mechanical analysis (DMA) and X-ray diffraction (XRD), and the relationships among the heating rate, structure evolution and mechanical properties of the final PI fibers are determined.

## 2. Materials and Methods

### 2.1. Materials

The BPDA and PDA were purchased from the Shanghai Research Institute of Synthetic Resins, Shanghai, China. The BPDA was dried at 200 °C for 20 h in a nitrogen atmosphere (High Purity, Juyang Gas, Changchun, China). The PDA was purified by sublimation. The DMAc was purchased from Tianjin Fine Chemical Company, Tianjin, China, and used according to kit instructions.

### 2.2. Preparation of PAA Fibers

**Scheme 1:**
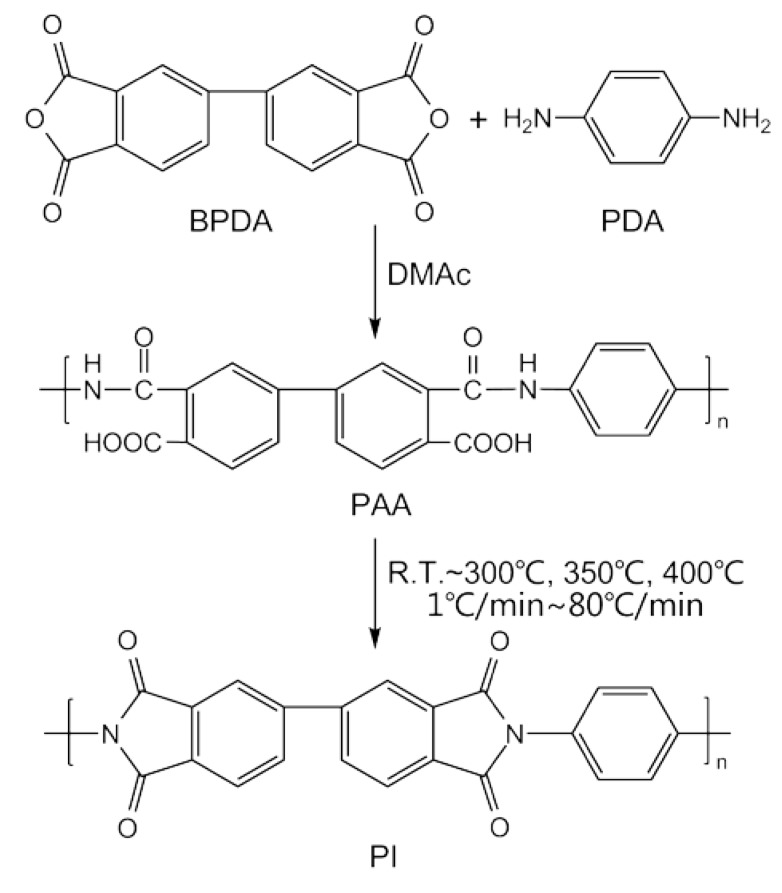
Synthesis of the BPDA-PDA polyamic acid and polyimide fibers.

The BPDA-PDA PAA was synthesized in a dried DMAc solvent by mixing BPDA and PDA in a molar ratio of 1.01/1.00. The PDA monomer was dissolved in DMAc first, and BPDA monomer was then added in a nitrogen atmosphere. After stirring at 0 °C for 8 h, a yellow viscous solution with 15 wt % solid content was obtained. The intrinsic viscosity of the PAA was measured as 1.25 dL/g by Ubbelohde viscometer (the capillary diameter is 0.57 mm) at 30 °C through a dilution method. This PAA solution was filtered and degassed before fiber spinning. As-spun PAA fibers were prepared via dry-jet wet-spinning technology, by extruding the PAA solution through a spinneret (50 holes, 120 μm) into air first, and then drawn into a coagulation bath. The air gap between the spinneret and the coagulation bath was approximately 5 cm. The coagulation bath was a mixture of water and DMAc (*v/v* = 5/5). After several times being washed by deionized water, the as-spun PAA fibers were dried in an air oven at 80 °C for 12 h. The synthetic route of the PAA and PI fibers is shown in Scheme 1.

### 2.3. Preparation of PI Fiber

The PI fibers were obtained under different heating rates by utilizing TGA equipment (Q50, TA Instruments, New Castle, DE, USA) as a furnace. The PAA fibers were treated using TGA at heating rates of 1, 2, 5, 10, 20, 40 and 80 °C/min from room temperature to 300, 350 and 400 °C.

### 2.4. Characterization

**Figure 1:**
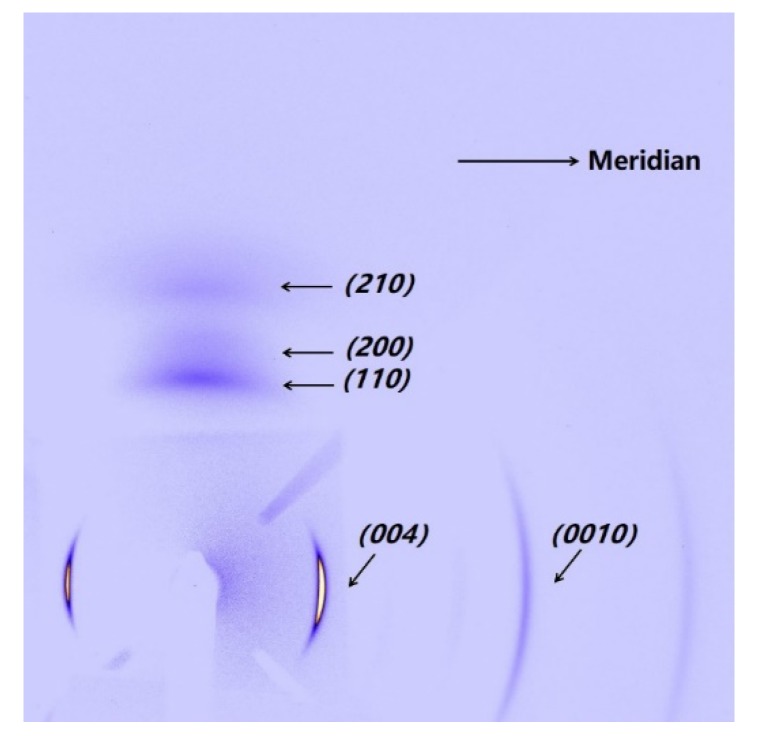
2D WAXD patterns of PI fiber (fiber treated at 40 °C/min from room temperature to 400 °C as an example). The steaks of (004) and (0010) and the streaks of (110), (200) and (210) are on the meridian and equatorial directions of the PI fiber, respectively.

The imidization process of the PAA fibers was characterized on the basis of the curve trends detected by TGA. The imidization degrees of fiber samples was calculated from the FTIR spectra obtained through a FTIR spectrometer (Bruker Vertex 70, Billerica, MA, USA) in an attenuated total reflection mode. The bands at 1552 and 1380 cm^−1^, which respectively represent the absorption of benzene ring and imide N–C stretching of PI, were chosen to estimate the imidization degrees of the fibers. DMA (SDTA861e, Mettler Toledo, Schwerzenbach, Zürich, Switzerland) was carried out on the PAA fibers in tension mode at a frequency of 1 Hz. A series of heating rates (1, 2, 3, 3.5, 4, 5, 8, 10, 16 and 20 °C/min) were adopted for DMA characterization. The densities of the PI fibers were measured by a buoyancy method [20]. The XRD (SmartLab, Rigaku, Akishima-shi, Tokyo, Japan) patterns were obtained along and perpendicular to the fiber axis. The PI fibers were twisted on the particular holder designed by our laboratory, enabling the fiber to be characterized quantitatively. The area and location of certain diffraction peaks were studied and analyzed with the Origin 8.0 (OriginLab Corporation, Northampton, MA, USA) and PeakFit 4.0 software (Systat Software, San Jose, CA, USA). The 2D wide-angle X-ray diffraction (WAXD) patterns of the PI fiber obtained from the Shanghai Synchrotron Radiation Facility are shown in Figure 1. The most isolated (004) streaks on the 2D WAXD patterns were determined, and Hermans’ orientation factor (*f*_c_) was calculated to characterize the preferred orientation of crystals in the fiber [20]. The tensile properties of all of the PI fibers were characterized by an extensometer (XQ-1C, New Fiber Instrument, Shanghai, China) with a cross-head speed of 5 mm/min. Each sample was tested 30 times, and the average value was adopted as the final result.

## 3. Results

### 3.1. Imidization Process as Detected by TGA

**Figure 2:**
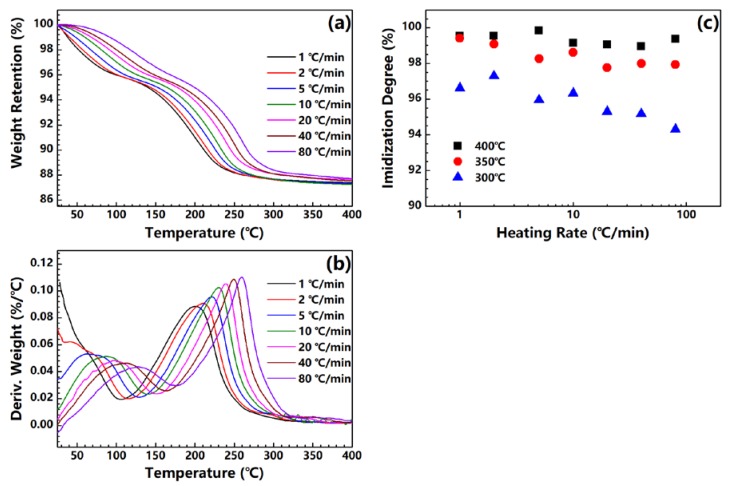
(**a**) TGA and (**b**) DTG curves of PAA fibers under different heating rates; (**c**) imidization degree of PI fibers obtained at 300 °C, 350 °C and 400 °C under different heating rates.

Figure 2a shows the TGA curves of the PAA fiber treated under different heating rates. Two steps of weight loss can be observed among the samples. The first step can be assigned to the evaporation of the absorbed water in the PAA fiber, while the second step originates from the water produced by the imidization reaction. As the heating rate increases, the TGA curve shifts to a high temperature. An imidization reaction is expected to occur in the high temperature range. The derivatives of the TGA curves are plotted in Figure 2b. The maximum of the DTG curve also shifts to the high temperature, and increases to a large value as the heating rate is increased. This trend implies that the reaction rate of imidization in the PAA fiber increases when the heating rate is increased. Figure 2c shows the plot of the imidization degrees of the PI fibers obtained at 300, 350 and 400 °C under different heating rates. Almost all of the samples exhibit imidization degrees of higher than 95% regardless of their respective end temperatures, even for the fibers obtained at 300 °C; this finding is consistent with that of our previous research [20]. The imidization degree evidently decreases with the increase in heating rate, especially for fibers with end temperatures of 300 °C. Under such high heating rates, the fibers do not have enough time to realize full imidization. On the basis of this trend, for fibers treated under different high heating rates, a high-end temperature is needed to ensure complete imidization.

### 3.2. DMA Measurement

**Figure 3:**
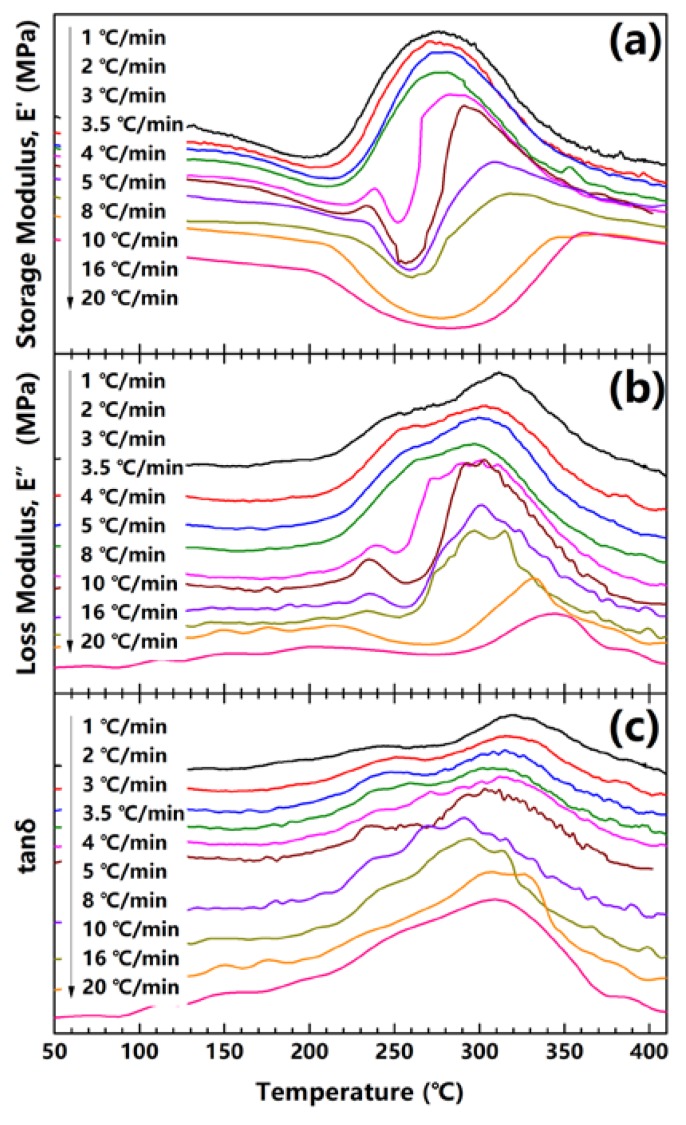
(**a**) Storage modulus; (**b**) loss modulus; and (**c**) tanδ of PAA fibers with temperatures under different heating rates.

The PAA fibers are characterized in DMA tensile mode under different heating rates, ranging from 1 °C/min to 20 °C/min. The curves of the storage modulus (E′), loss modulus (E″) and tanδ with temperatures are plotted in Figure 3a–c. For instance, for PAA fibers treated at 1 °C/min, E′ decreases slightly from 125 to 200 °C, which aligns with imidization reaction. Then, as the temperature exceeds 200 °C, E′ significantly increases at the beginning of crystallization in the fiber. When the temperature is higher than 275 °C, E′ remarkably decreases due to the glass transition of the amorphous phase in the PI fiber. Subsequently, the experimental heating rate is increased from 1 to 3.5 °C/min. The E′ curve shifts slightly to the high temperatures, but shows a similar behavior. 

When the heating rate exceeds 3.5 °C/min, the E′ curve rapidly drops to approximately 240 °C. This E′ drop expands significantly with the increase in heating rate, and fully offsets the increase in E′ when the heating rate exceeds 10 °C/min. The drop in E′ is clearly caused by the *T*_g_ of the fiber, which consists of non-fully imidized PAA-PI chains. This kind of *T*_g_ is somewhat dependent on the experimental heating rate and the imidization degree of the non-fully imidized fiber, as *T*_g_ may increase with the rise in the imidization degree when the flexible PAA chains are converted into rigid PI chains. At low heating rates, the increase in *T*_g_ generally matches the experimental temperature, and no distinguishable *T*_g_ is likely to be detected on the E′ curve. By contrast, at the high heating rate range, the experimental temperature increases rapidly, and is expected to surpass the *T*_g_ of the fibers at low temperatures. As shown in Figure 3a, *T*_g_ emerges at a much lower temperature under high heating rates. On the basis of Figure 2a, we can further conclude that the experimental temperature can surpass the *T*_g_ with a low imidization degree at high heating rates. Moreover, as the experimental temperature exceeds the *T*_g_ of the fiber, the polymer chains can achieve much higher mobility. Under high heating rates, imidization reaction and crystallization are expected to occur simultaneously.

### 3.3. Density Measurement

**Figure 4:**
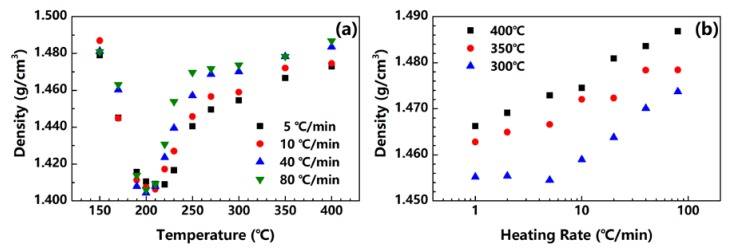
(**a**) Density change in fibers under different heating rates; (**b**) density of PI fibers obtained at 300, 350 and 400 °C under different heating rates.

Figure 4 shows the densities of the fibers treated at 5, 10, 40 and 80 °C/min. All of the fibers treated under different heating rates show a similar behavior. At first, the fiber density decreases because of the imidization reaction, which releases water into the fiber. Then, the density starts to increase as the crystals substantially form. Density is dependent on both imidization and crystallization during treatment, and the minimum of the fiber density needs to be observed. As shown in Figure 4a, for fibers treated at 5, 10, 40 and 80 °C/min, the temperatures of the density minima are 220, 210, 200 and 200 °C, respectively. Compared with Figure 2c, the imidization conversion at the density minima in Figure 4a are 60%, 39%, 18% and 15%, respectively. The crystallization in the fiber can start at low temperatures and imidization conversions under a high heating rate. In other words, when PAA fibers are heated at a high rate, imidization and crystallization may occur simultaneously, which is somewhat consistent with the DMA conclusion. Furthermore, for fibers treated with high heating rates, the fiber density significantly increases after the minimum, and finally reaches a much higher value. This finding indicates that the formation of crystals in the fiber is more vigorous at high heating rates than that of low heating rates. This phenomenon is believed to offer more evidence proving that the mobility of polymer chains is much higher under high heating rates, as discussed previously with the topic on DMA. 

Figure 4b shows the densities of the fibers treated under different heating rates and the varying end temperatures. For the PI fibers obtained at 300, 350 and 400 °C, their densities monotonically increase with the heating rate from 1.46 to 1.47 g/cm^3^, from 1.46 to 1.48 g/cm^3^ and from 1.47 to 1.49 g/cm^3^, respectively. This finding proves that PI fibers can obtain high crystallinity under high heating rates.

### 3.4. Crystallization Evolution and Orientation by XRD Patterns

**Figure 5:**
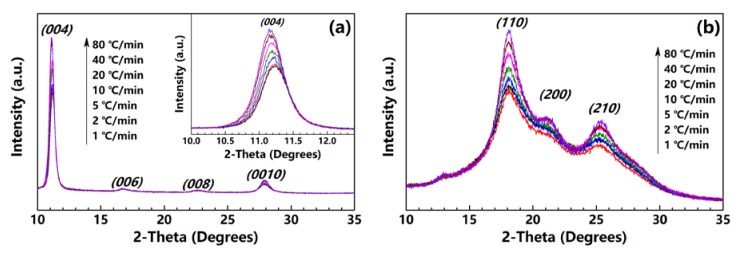
XRD patterns along the (**a**) meridian and (**b**) equatorial directions of fibers obtained at 400 °C under different heating rates.

**Figure 6:**
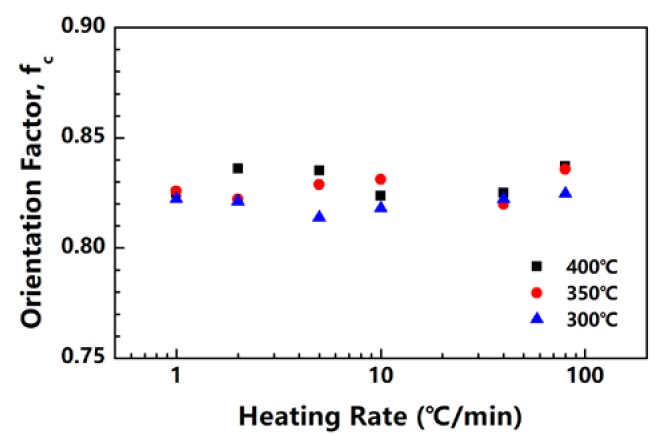
Crystal orientation factors of PI fibers obtained under different heating rates.

The PI fibers obtained at 300, 350 and 400 °C under different heating rates are characterized by XRD along and perpendicular to the fiber axis. Figure 5a,b show the meridian and equatorial diffraction patterns of the PI fibers obtained at 400 °C (sampled case). The orthorhombic unit cell [21] is considered; for all fiber samples, the meridian peaks at approximately 11.2°, 16.9°, 22.8° and 28.1° can be indexed as (004), (006), (008) and (0010), and the equatorial peaks at approximately 18.2°, 20.9°, and 25.3° can be assigned to (110), (200) and (210). The (00l) and (hk0) diffraction peaks appear at the meridian and equatorial directions, respectively. Such locations imply a preferred orientation for the crystals in the fiber. Figure 6 shows the Hermans’ orientation factors for the fibers calculated using the (004) streaks on 2D WAXD profiles. The values of *f_c_* are similar, at approximately 0.82 regardless of the heating rate, indicating similar crystal orientations in the PI fibers.

**Figure 7:**
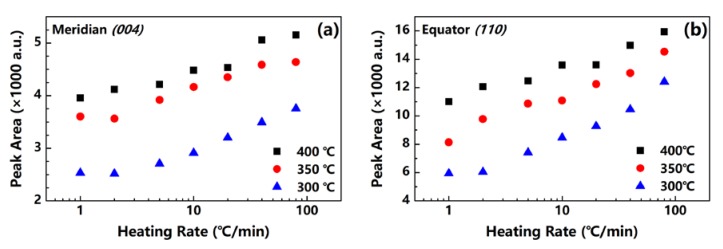
Areas of (**a**) (004) and (**b**) (110) diffraction peaks of PI fibers obtained under different heating rates.

Figure 7 shows the values of the (004) and (110) peak areas at the meridian and equatorial directions of the fibers obtained at 300, 350 and 400 °C. The diffraction intensity increases significantly with the increase in the heating rate, regardless of the end temperature. Considering the similarity in *f_c_* values, we assume that the increase in diffraction intensity is caused only by the formation of crystals in the fiber, further demonstrating the promotion of high heating rates in relation to the crystallinity of the PI fibers. The result is in agreement with the density results mentioned above.

In addition, as shown in the meridian diffraction patterns (inset in Figure 5a), the (004) peak shifts significantly to a much smaller angle with the increase in the heating rate, indicating an increase in the axial distance for the crystal lattice. Table 1 lists the diffraction angles and full-width-of-half-maximum of the (004) peaks of all of the samples, and the calculated axial d-spacings and crystal sizes based on the Bragg and Scherrer equations. As the heating rate increases from 1 to 80 °C/min for the fibers obtained at 300, 350 and 400 °C, the calculated axial d-spacings increase significantly from 31.50 to 31.70 Å, from 31.50 to 31.76 Å and from 31.56 to 31.87 Å, respectively. The axial crystal sizes also remarkably increase from 173.08 to 184.78 Å, from 165.05 to 172.58 Å and from 173.36 to 181.08 Å. Obviously, PI fibers obtained under a much higher heating rate exhibit a much larger axial d-spacing and crystal size. This indicates that under a high heating rate, PI chains cannot perfectly arrange themselves onto the lattice. This is because the PI chains may be frozen quickly after the heating treatment, and do not have enough time to move or adjust to the conformation to form a perfect lattice. So, much higher residual stress may be induced in the fiber under a high heating rate, subsequently directly affecting the mechanical properties.

### 3.5. Mechanical Properties

**Figure 8:**
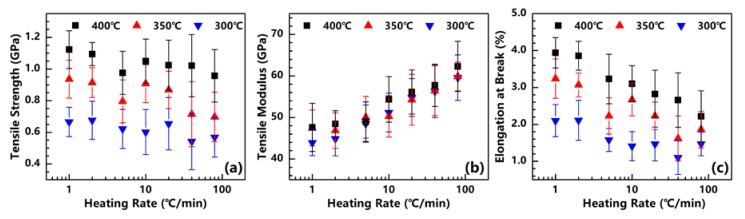
Tensile strength (**a**), Tensile modulus (**b**), and Elongation at break (**c**) of PI fibers obtained under different heating rates.

The mechanical properties of the PI fibers obtained under different heating rates are measured. For each heating rate, the test sample of a PI fiber is taken under the different temperatures of 300, 350 and 400 °C. The tensile strength, tensile modulus and elongation at each break of each sample are plotted in Figure 8.

As reported in our previous research [20], the tensile strength of PI fibers can be improved significantly with the increase in crystallinity. As for the PI fibers obtained under different heating rates, the above-mentioned density and XRD results demonstrate that the crystallinity of fibers can be increased by significantly raising the heating rate. However, the tensile strength shown in Figure 8a slightly declines with the increase in the heating rate. The tensile strengths of the PI fibers obtained at 300, 350 and 400 °C decrease from 0.67, 0.94 and 1.12 GPa at 1 °C/min to 0.57, 0.70 and 0.96 GPa at 80 °C/min. We assume that this result is related to residual stress, which rises with the increase in the heating rate. Although crystallinity can be promoted with high heating rates, a much higher residual stress is also produced, causing the fiber to be fractured much more easily during measurement. In addition, tensile strength can also be significantly promoted by treatment temperature. As a sample case, the PI fibers are treated at 1 °C/min, with the tensile strength shifting from 0.67 to 1.12 GPa, and the treatment temperature increasing from 300 to 400 °C. As emphasized in our previous study [20], high treatment temperatures may effectively eliminate the non-imide units of polymer chains. Obviously, a few defects in fibers under high treatment temperatures is advantageous in ensuring the good tensile strength of these fibers.

Figure 8b presents the tensile moduli of the obtained PI fibers. For the PI fibers obtained at 300, 350 and 400 °C, as the heating rate increases from 1 to 80 °C/min, the tensile moduli remarkably grow from approximately 45 to 60 GPa. General knowledge dictates that raising the crystallinity will affect the tensile modulus, given the enhanced stiffness of a fiber. Our finding proves that the crystallinity of the PI fiber can be promoted by both the heating rate and treatment temperature. The tensile modulus is significantly dependent on heating rate, but seems non-significantly dependent on the treatment temperature. This finding indicates that the behavior of the tensile modulus cannot be determined by simply referring to the crystallinity. We assume that as the PAA fibers are treated under high heating rates (attributable to PI chains possessing high mobility, except for those with high crystallinity), the PI chains can also arrange themselves to form a more continuous crystal region. In other words, the tensile modulus depends on the size of the crystal region and not on crystallinity. Similar to the findings in our previous research [20], when the PAA fibers are isothermally treated in a muffle furnace at temperatures of 300, 350 and 400 °C, the tensile moduli of the obtained PI fibers are independent of the treatment temperature, as proven by their similar values. This present work provides a further explanation of the previous study’s result, i.e., although the treatment temperatures differ, the PAA fibers experience a similar heating rate in a muffle furnace. The sizes of the crystal regions in the PI fiber are identical, and thus the PI fibers exhibit similar tensile moduli.

Figure 8c shows the elongation at the break of the PI fibers. For all samples obtained at 300, 350 and 400 °C, the elongation at the break decreases significantly at the heating rates of 2.1%, 3.2% and 3.9% to 1.5%, 1.9% and 2.2%. As a common behavior of the fibers, the elongation at the break will decrease with the increase in tensile modulus, as an increase in the stiffness of the fiber may restrict its ability to elongate. Moreover, the elongation at the break increases significantly with the rise in treatment temperature. The same reason has been given for tensile strength, with the high treatment temperature possibly eliminating the defects of a fiber.

### 3.6. Isothermal Annealing after Treatment

Although increasing the heating rate can effectively promote the crystallinity of PI fibers and remarkably enhance their respective tensile moduli, the tensile strength does not increase, and instead slightly decreases due to the high residual stress under high heating rates. A thermal annealing process is necessary to reduce the residual stress. Here, the PI fiber obtained at 80 °C/min-400 °C is annealed at 400 °C under different times. The annealed fiber is characterized by XRD and tensile testing for further study. 

Table 2 lists the diffraction angles, full-width-at-half-maximum values, axial d-spacings and crystal sizes of the annealed PI fibers. As annealing time increases from 0 to 25 s, the axial d-spacing and crystal size both remarkably decrease from 31.87 and 181.08 Å to 31.73 and 154.80 Å. This result proves that when the PI fiber is annealed, the crystal lattice becomes highly compact, indicating that the residual stress in the fiber has been greatly reduced. Moreover, it should be noticed that the above process is only carried out in less than 25 s, which is a rather short time.

**Figure 9:**
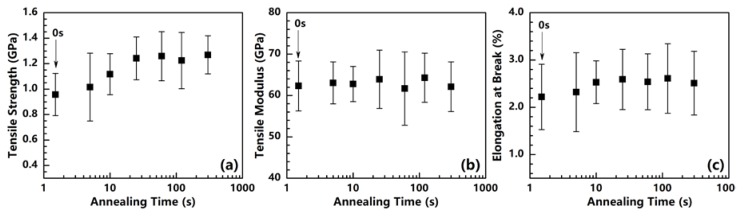
Tensile strength (**a**), Tensile modulus (**b**), and Elongation at break (**c**) of PI fibers annealed at 400 °C under different times.

The mechanical properties of the annealed PI fibers are plotted in Figure 9. From 0 to 25 s, the tensile strength and elongation at the break simultaneously increase, the tensile strength increases from 0.96 to 1.24 GPa, and the elongation at the break slightly increases from 2.2% to 2.6%. This result proves that annealing can reduce residual stress and improve both tensile strength and elongation at the break. As for the tensile modulus, this study finds it somewhat insensible for the annealing process to remain at the constant value of approximately 62 GPa, regardless of the annealing time.

## 4. Conclusions

In this study, PAA fibers deriving from BPDA and PDA were successfully prepared via a conventional dry-jet wet-spinning process. The PAA fibers are treated from room temperature under different heating rates (1 to 80 °C/min) to different end temperatures (300, 350 and 400 °C) to obtain PI fibers. The imidization and crystallization processes during the treatment are characterized by TGA, DMA, XRD and density measurement. Under a low heating rate, the crystallization process lags behind the imidization process. As the heating rate increases, the imidization and crystallization processes tend to occur simultaneously. The polymer chains in fiber exhibit higher mobility at a higher heating rate. The crystallinity of PI fiber can be improved by increasing the heating rate or the end temperatures. The tensile modulus of PI fiber increases monotonically with the rise in the heating rate. As the heating rate increases from 1 to 80 °C/min, the tensile modulus increases from approximately 45 to 60 GPa regardless of the end temperatures. According to this fact, it is very interesting to find that the tensile modulus is not determined by crystallinity. We assume that it is the size of crystal region in fiber, not the crystallinity, that determines the tensile modulus. The tensile strength of PI fibers shows a reduction when increasing the heating rate. As the heating rate increases from 1 to 80 °C/min, the tensile strength of the PI fibers obtained at 300, 350 and 400 °C decreases from 0.67, 0.94 and 1.12 GPa to 0.57, 0.70 and 0.96 GPa, respectively. Obviously, it is the residual stress in the fiber, which is notably enhanced under a high heating rate, that makes it more easy to break. An annealing process under a high temperature proves effective to reduce residual stress in a fiber. When a PI fiber treated under 80 °C/min from room temperature to 400 °C is annealed at 400 °C, the fracture strength exhibits a notable improvement from 0.96 to 1.24 GPa in just 25 s. This demonstrates that high temperature annealing is necessary and effective to improve the tensile strength in PI fiber fabrication.

## Figures and Tables

**Table 1:** D-spacing, crystal size and orientation factors calculated using the (004) diffraction for PI fibers obtained at 300, 350 and 400 °C under different heating rates.

End Temperature (°C)	Heating Rate (°C/min)	2-Theta (Degrees)	FWHM (Degrees)	d-spacing (Angstrom)	Crystal Size (Angstrom)
400	1	11.22	0.46	31.56	173.36
	2	11.21	0.45	31.59	175.08
	5	11.18	0.46	31.67	173.14
	10	11.16	0.44	31.73	181.12
	20	11.15	0.44	31.76	179.92
	40	11.14	0.43	31.79	184.91
	80	11.11	0.44	31.87	181.08
350	1	11.24	0.49	31.50	165.05
	2	11.22	0.48	31.56	163.55
	5	11.19	0.47	31.64	167.11
	10	11.19	0.47	31.64	167.40
	20	11.17	0.45	31.70	175.97
	40	11.16	0.45	31.73	174.83
	80	11.15	0.46	31.76	172.58
300	1	11.24	0.46	31.50	173.08
	2	11.23	0.45	31.53	176.27
	5	11.22	0.44	31.56	181.86
	10	11.20	0.43	31.62	182.40
	20	11.19	0.43	31.64	184.04
	40	11.18	0.49	31.67	184.08
	80	11.17	0.43	31.70	184.78

**Table 2:** D-spacing and crystal size calculated using the (004) diffraction for PI fibers annealed at 400 °C under different times.

Annealing Time (s)	2-Theta (Degrees)	FWHM (Degrees)	d-spacing (Angstrom)	Crystal Size (Angstrom)
0	11.11	0.44	31.87	181.08
5	11.12	0.44	31.84	179.42
10	11.14	0.47	31.79	167.97
25	11.16	0.51	31.73	154.80
60	11.16	0.52	31.73	151.82
120	11.16	0.51	31.73	154.80
300	11.16	0.52	31.73	151.82
